# Fluids and sepsis: changing the paradigm of fluid therapy: a case report

**DOI:** 10.1186/s13256-016-1191-1

**Published:** 2017-02-04

**Authors:** Hori Hariyanto, Corry Quando Yahya, Monika Widiastuti, Primartanto Wibowo, Oloan Eduard Tampubolon

**Affiliations:** 1 Department of Anesthesiology and Critical Care Medicine, 3rd floor, Siloam Hospitals Lippo Village, Jalan Siloam No. 6, Karawaci, 15811 Tangerang, Banten Indonesia; 2 Department of Anesthesiology, Faculty of Medicine, Universitas Pelita Harapan, Jalan Boulevard Jendral Sudirman, Lippo Karawaci, Tangerang, 15811 Indonesia

**Keywords:** Sepsis, Septic shock, Fluid management, Fluid overload, Geriatric, Case report

## Abstract

**Background:**

Over the past 16 years, sepsis management has been guided by large-volume fluid administration to achieve certain hemodynamic optimization as advocated in the Rivers protocol. However, the safety of such practice has been questioned because large-volume fluid administration is associated with fluid overload and carries the worst outcome in patients with sepsis. Researchers in multiple studies have declared that using less fluid leads to increased survival, but they did not describe how to administer fluids in a timely and appropriate manner.

**Case presentation:**

An 86-year-old previously healthy Sundanese man was admitted to the intensive care unit at our institution with septic shock, acute kidney injury, and respiratory distress. Standard care was implemented during his initial care in the high-care unit; nevertheless, his condition worsened, and he was transferred to the intensive care unit. We describe the timing of fluid administration and elaborate on the amount of fluids needed using a conservative fluid regimen in a continuum of resuscitated sepsis.

**Conclusions:**

Because fluid depletion in septic shock is caused by capillary leak and pathologic vasoplegia, continuation of fluid administration will drive intravascular fluid into the interstitial space, thereby producing marked tissue edema and disrupting vital oxygenation. Thus, fluids have the power to heal or kill. Therefore, management of patients with sepsis should entail early vasopressors with adequate fluid resuscitation followed by a conservative fluid regimen.

## Background

Fluid administration has been a topic of interest since the development of aggressive fluid resuscitation by the 2003 Surviving Sepsis Campaign [1]. Although it is believed that fluids play a vital part in sepsis management, recent studies of large-volume fluid administration have shown conflicting results. Authors of a systematic review on fluids in critically ill and injured patients reported data derived from 19,902 subjects to be conclusive regarding the harm of positive fluid balance. Patients with a positive cumulative balance of 6982 ± 5629 ml have a higher mortality rate than those patients with an overall cumulative fluid balance of 2449 ± 2965 ml by day 7 (24.7% vs. 33.2%, OR 0.42, 95% CI 0.32–0.55, *p* < 0.0001) [2].

Administration of intravenous fluid remains one of the most common therapies given to hospitalized patients; however, studies have shown that up to 20% of these patients are given inappropriate fluid therapy [3]. As subtle as it seems, fluid therapy is a double-edged sword that carries the potential either to reverse organ damage or to cause irreversible damage. Fluid resuscitation at the early stages of shock is necessary to reverse life-threatening conditions, but what happens after this stage has passed? Should fluid resuscitation be continued, or should fluids start to be tapered? Surely, fluid therapy cannot be applied as a one-size-fits-all solution.

With new insights into fluid administration and clinical outcome, perhaps the use of large-volume fluid resuscitation in the management of patients with sepsis ought to be reconsidered. How much fluid is needed in what amount of time, and what are the parameters for monitoring a safe and adequate fluid balance? In a review on intravenous fluid therapy, Hoste *et al*. divided fluid administration into four phases: resuscitation, optimization, stabilization, and evacuation (ROSE) [3]. In this case report, we describe the use of ROSE fluid management along with parameters for monitoring a safe and adequate fluid balance throughout the development of sepsis.

## Case presentation

**Fig. 1:**
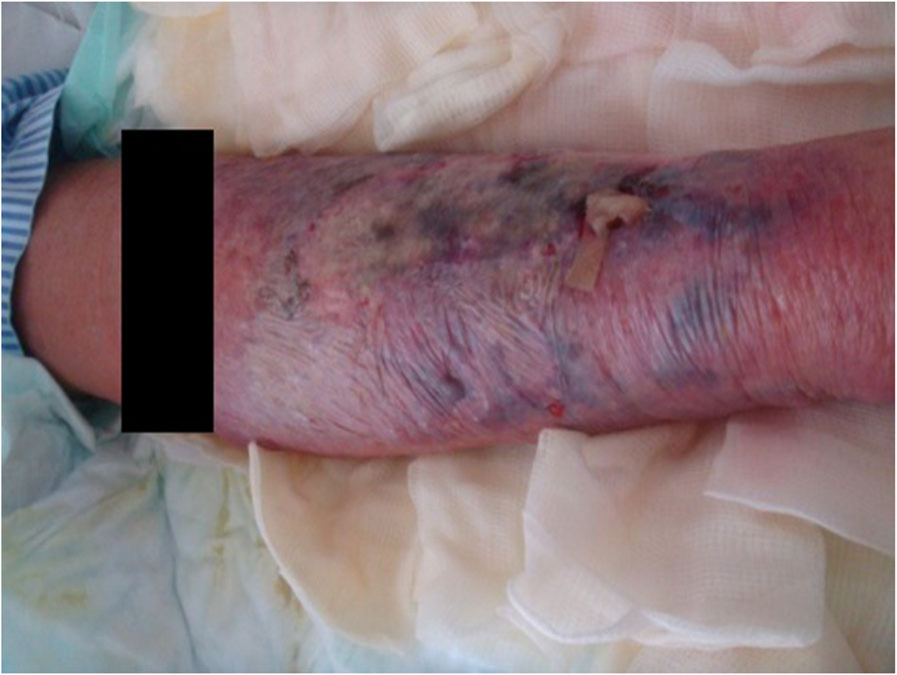
Necrotic and pustular tissue formation on the right arm upon intensive care unit admission

A previously healthy 86-year-old Sundanese man with no comorbidities was admitted to the general ward of our hospital with excruciating pain in his right hip and knee after a prior fall. Our patient weighed 65 kg and was 165 cm tall. On admission, he was fully alert, and the results of his radiologic investigations were normal. Intravenous analgesics and nerve blocks were administered, and the patient remained hospitalized for 12 days of nursing care. On the 12th day, intravenous catheter site induration and redness developed, which rapidly progressed to necrotic and pustular tissue formation within 12 h (Fig. 1). A wound culture was taken, and intravenous antibiotic therapy was promptly initiated; nevertheless, the patient’s condition worsened on day 14 of his hospitalization, and he became lethargic.

Our patient was moved to the high-care unit (HCU) with the following hemodynamic parameters: blood pressure 107/46 mmHg, mean arterial pressure (MAP) 66 mmHg, heart rate 88 beats per minute (bpm), respiratory rate 21 breaths per minute, and oxygen saturation 100% through a 5-L nasal cannula. He remained afebrile. A complete blood examination revealed hemoglobin (Hgb) 1.94 g/dl (reference range 11.70–15.50 g/dl), hematocrit (Hct) 32.35% (reference range 35.00–47.00%), white blood cell count (WBC) 20,550/mm^3^ (reference range 3600–11,000/mm^3^), platelets (Plt) 137,700/μl (reference range 150,000–440,000/μl), C-reactive protein 199.90 mg/L (reference range 0.00–3.00 mg/L), and procalcitonin (PCT) 37.00 ng/ml (reference range <0.5 ng/ml). Other levels recorded were alanine aminotransferase 13 U/L (reference range 0–55 U/L), aspartate transaminase 12 U/L (reference range 5–34 U/L), urea 126.0 mg/dl (reference range <50 mg/dl), creatinine 2.42 mg/dl (reference range 0.5–1.1 mg/dl), Na^+^ 131 mEq/L (reference range 135–145 mEq/L), K^+^ 6.2 mEq/L (reference range 3.5–5 mEq/L), Cl^−^ 101 mEq/L (reference range 96–110 mEq/L), and random blood glucose 114 mg/dl.

A diagnosis of necrotizing fasciitis with sepsis, stage 2 acute kidney injury, and hyperkalemia was made. One gram of intravenous cefoperazone twice daily and 400 mg of moxifloxacin once daily were given. The patient’s hyperkalemia was treated using 25 U of insulin and 100 ml of 40% dextrose solution for 2 h. A nasogastric tube was inserted, and the patient’s stomach was decompressed. A central venous catheter was inserted, and cultures from blood, urine, and sputum were taken.

Nevertheless, the patient’s condition worsened. He became unresponsive with a respiratory rate of 38 breaths per minute and prominent use of accessory muscles. His oxygen saturation was 88% with a 15-L non-rebreathing mask; his central venous pressure (CVP) was 5 mmHg; his blood pressure was 90/60 mmHg (MAP 70 mmHg); and he had an electrocardiographic reading of atrial fibrillation with rapid ventricular response and a heart rate of 140–160 bpm. Arterial blood gas analysis revealed respiratory acidosis with pH 7.029, partial pressure of carbon dioxide (pCO_2_) 77.9 mmHg, partial pressure of oxygen (pO_2_) 94 mmHg, HCO_3_^−^ 20.9 mEq/L, base excess −10 mEq/L, and serum lactate 3.3 mmol/L (reference range <0.6–2.2 mmol/L). The patient’s blood pressure continued to fall and reached 60/30 mmHg (MAP 40 mmHg), followed by multiple episodes of bradycardia from 140 bpm to 70 bpm despite administration of 500 ml of colloid and 100 ml of 20% albumin. Hence, noradrenaline at 0.5 μg/kg/minute and dobutamine at 10 μg/kg/minute were initiated. In the HCU, the patient received a total fluid input of 4644 ml with urine output of 55 ml/h and fluid balance of +3540 ml/20 h.

**Fig. 2:**
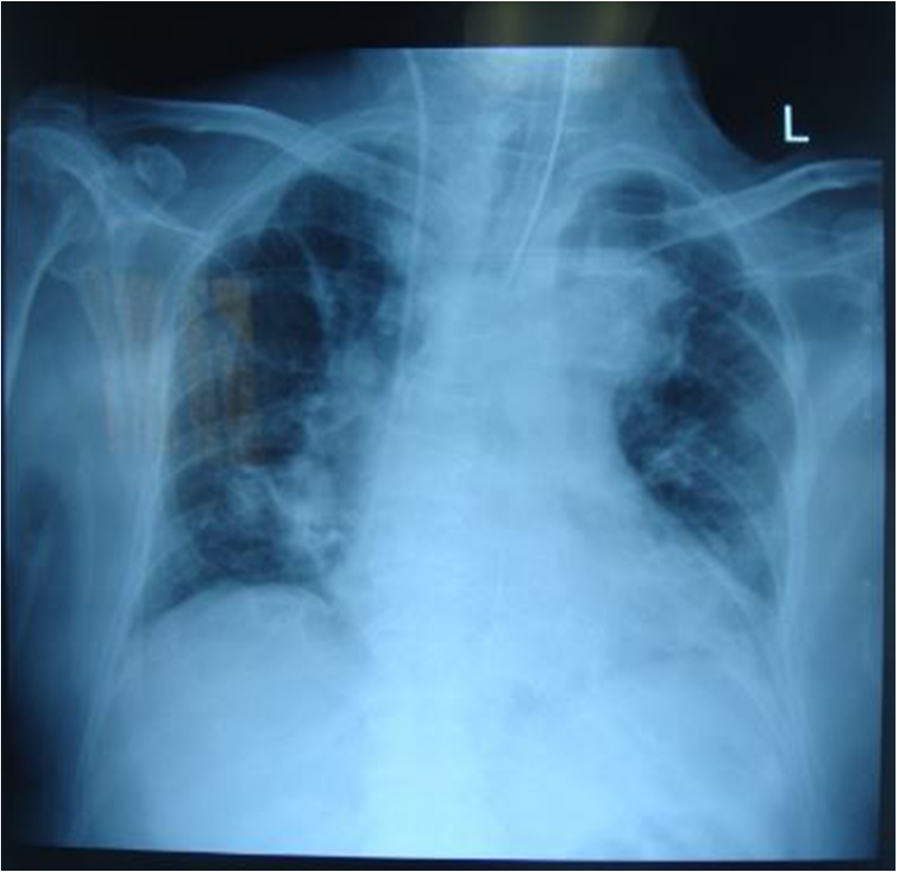
Chest x-ray taken on initial intensive care unit admission

The patient was promptly transferred to the intensive care unit (ICU), where he was intubated and mechanically ventilated. He was placed on adaptive support ventilation mode with a positive end-expiratory pressure of 5 cmH_2_O and a fraction of inspired oxygen of 0.5. At this time, his blood pressure plummeted to 80/50 mmHg (MAP 60 mmHg), and his CVP was 16 mmHg. Noradrenaline was increased to 0.8 μg/kg/minute and dobutamine to 3 μg/kg/minute, to which he responded. His blood pressure was maintained at 115/60 mmHg (MAP 78 mmHg); his heart rate was 110–120 bpm; and his CVP was 12 mmHg. Two hours postintubation, his blood gas analysis revealed pH 7.28, pCO_2_ 39.6 mmHg, pO_2_ 112.5 mmHg, HCO_3_^−^ 19.1 mEq/L, base excess −6.9 mEq/L, and a lactate level decreasing to 2.27 mmol/L. A chest x-ray revealed patchy infiltrates on the lower lung regions with a cardiothoracic ratio of 61% (Fig. 2), and echocardiography revealed an ejection fraction of 67% with no ventricular wall motion abnormalities.

Continuous analgesia and sedation with morphine and midazolam infusion were administered, and the patient’s vital signs stabilized. A repeat blood workup revealed insignificant changes except for urea and creatinine increasing to 159.5 mg/dl and 2.74 mg/dl, respectively. The patient’s PCT levels spiked to 97.60 ng/ml, and antibiotics were switched to meropenem 1 g every 8 h, moxifloxacin 400 mg once daily, and 200 mg fluconazole twice daily. At that time, our patient received 1000 kcal/500 ml of parenteral nutrition through intermittent bolus nasogastric feeding tubes.

On the second day, the results of a wound culture revealed the growth of *Streptococcus pyogenes*, and meropenem was changed to 400 mg of teicoplanin daily along with moxifloxacin, based upon antibiotic sensitivity results. Cultures from the patient’s blood and urine revealed no growth, whereas a sputum culture revealed growth of *Candida albicans*, and a fluconazole regimen was resumed. The patient’s blood gas analysis normalized with pH 7.38, pCO_2_ 40.6 mmHg, pO_2_ 138.8 mmHg, HCO_3_^−^ 16.6 mEq/L, base excess −3.9 mEq/L, and serum lactate 1.3 mmol/L. His blood pressure was stable at 110/50 mmHg (MAP 70 mmHg); his heart rate was 100–120 bpm with atrial fibrillation; and his CVP was 12 mmHg. Intravenous amiodarone at 150 mg for 10 minutes was given, followed by a continuous infusion of 150 mg for 12 h.

Wound debridement and necrotomy were performed on the second day. However, 1 h postdebridement, the patient’s blood pressure plummeted to 50/30 mmHg (MAP 38) with a heart rate of 100 bpm. A bolus of 100 ml of normal saline was given along with noradrenaline at 0.8 μg/kg/minute and epinephrine at 8 μg/kg/minute. The amiodarone infusion was stopped. The patient’s vital signs responded progressively, and epinephrine was slowly tapered and then completely discontinued after 2 h. Maintenance fluids were given at 40 ml/h normal saline with a total daily fluid input of 3850 ml, diuresis of 70 ml/h, and a daily fluid balance of +1255 ml.

On the third day, the patient’s mental status improved dramatically; he was able to respond to instructions, and his vital signs remained within normal limits. The ventilator mode and settings remained unchanged, and the patient was actively triggering breaths with good ventilator synchrony. A complete blood examination revealed Hgb 9.9 g/dl (reference range 11.70–15.50 g/dl), Hct 24.5% (reference range 35.00–47.00%), WBC 24,190/mm^3^ (reference range 3600–11,000/mm^3^), and a PCT level decreasing to 83.46 ng/ml. His coagulation profile revealed Plt 149,000/μl (reference range 150,000–440,000/μl) with a prothrombin time (PT) of 13.60 seconds, international normalized ratio (INR) of 1.15, activated partial thromboplastin time (aPTT) of 55.40 seconds, and D-dimer of 5.36 ng/ml. His urea decreased slightly to 151.7 mg/dl (reference range <50 mg/dl); his creatinine was 1.84 mg/dl; and his serum albumin was 2.88 mg/dl (reference range 3.5–5.3 mg/dl). Enteral nutrition was resumed because no residual gastric fluid was noted, and the maintenance fluid used was normal saline at 20 ml/h with noradrenaline tapered to 0.01 μg/kg/minute. Total daily fluid input was 2198 ml with diuresis of 91 ml/h and a fluid balance of −967 ml.

On the fourth day, the vasopressor infusion was discontinued. The patient remained afebrile and responsive; hence, weaning from mechanical ventilation was initiated. His vital signs remained stable throughout the weaning process, with a blood pressure of 110/70 mmHg (MAP 83 mmHg), heart rate of 85–90 bpm, and CVP of 9 mmHg. His physical examination revealed clear lung sounds confirmed by a clear chest x-ray, and the results of his arterial blood gas analysis were within normal limits. The maintenance fluid used was normal saline at 40 ml/h with a total daily fluid input of 2610 ml, diuresis of 100 ml/h, and a fluid balance of −765 ml.

**Fig. 3:**
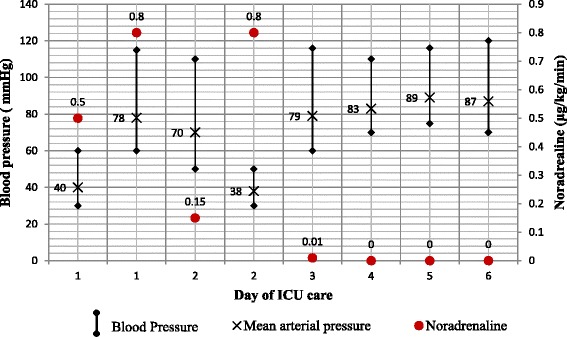
Daily mean arterial pressure and vasopressor dose. *ICU* Intensive care unit

On the fifth day, the patient was extubated. His vital signs remained stable 1 h postextubation with a respiratory rate of 18 breaths per minute and CVP of 10 mmHg, and his arterial blood gas analysis showed pH 7.428, pCO_2_ 26.4 mmHg, pO_2_ 173.1 mmHg, HCO_3_^−^ −17.8 mEq/L, and base excess −5.1 mEq/L. A repeat blood workup revealed Hgb 10.28 g/dl, Hct 31%, WBC 17,380/mm^3^, and Plt 114,000/μl. Other readings were PT 14.60 seconds, INR 1.24, aPTT 43.80 seconds, and D-dimer 5.90. The patient’s urea level was 130.9 mg/dl, and his creatinine level was 1.24 mg/dl. His nasogastric tube was withdrawn, and he was started on oral feeding. Normal saline was given at 20 ml/h with a total daily fluid input of 2562 ml, diuresis of 148 ml/h, and a daily fluid balance of −1998 ml (Fig. 3).

On the sixth day, he was discharged to the general ward. Normal saline was given at 20 ml/h with a total daily fluid input of 1858 ml, diuresis of 143 ml/h, and a daily fluid balance of −2537 ml (Table 1). An order to complete his 10-day course of intravenous moxifloxacin and his 14-day course of intravenous teicoplanin was completed, and he was discharged to home after 10 days of care in the general ward, without any negative sequelae.

**Table 1:** Daily vital signs, vasopressors, and fluids

	Day 1 HCU (2:00 a.m.)	Day 1 ICU (7:00 a.m.)	Day 2 ICU (7:00 a.m.)	Day 2 postdebridement (3:00 p.m.)	Day 3 ICU (7:00 a.m.)	Day 4 ICU (7:00 a.m.)	Day 5 ICU (7:00 a.m.)	Day 6 ICU (7:00 a.m.)
Vital signs								
Blood pressure, mmHg	60/30	115/60	110/50	50/30	116/60	110/70	116/75	120/70
Heart rate, beats/minute	140	120	120 (atrial fibrillation)	100	95	90	85	80
Mean arterial pressure, mmHg	40	78	70	38	79	83	89	87
Respiratory rate, breaths/minute	38	30	20–25	40–45	15–18	16–20	18	16
Central venous pressure, mmHg	18	12	12	12	10	9	9	8
Lactic acid, mmol/L	3.3	2.27	1.3	2.56	1.2	Not available	Not available	Not available
Vasopressors								
Norepinephrine, μg/kg/minute	0.5	0.8	0.15	0.8	0.01	None	None	None
Epinephrine, μg/kg/minute	None	None	None	8 (titrated and discontinued after 2 h)	None	None	None	None
Dobutamine, μg/kg/minute	10	3	3	None	None	None	None	None
Fluids	20 h	4 h		24 h	24 h	24 h	24 h	24 h
Input, ml	4644	100		3850	2198	2610	2562	1858
Urine output, ml/h	55	20		70	91	100	148	143
Fluid balance, ml	+3540	+34		+1255	−967	−765	−1998	−2537

*HCU* High-care unit, *ICU* Intensive care unit

**Fig. 4:**
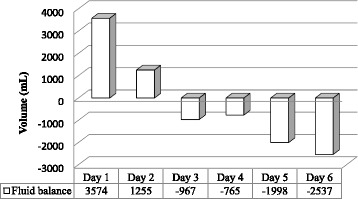
Daily fluid balance

Throughout his stay, our patient received metoclopramide, proton pump inhibitors, and daily nebulized salbutamol and mucolytic agents. Endotracheal suctioning was carried out as needed through a closed system device. Additionally, deep vein thrombosis prophylaxis was carried out using compression stockings and an intermittent pneumatic device. The wound site was cared for meticulously with daily dressing changes, and healing progressed significantly. Daily fluid balance was calculated by accounting for fluid input as all fluids administered through intravenous or nasogastric routes and metabolism products, which were one-third the value of insensible water loss (325 ml/day). Fluid output was counted as fluids collected from urine, wound drainage, nasogastric fluids, and insensible water loss, which was calculated at 15% of body weight in milliliters (975 ml/day) (Fig. 4).

## Discussion

Necrotizing fasciitis is a rapid and progressive necrotizing process involving the subcutaneous fat, superficial fascia, and superficial deep fascia [4]. The diagnosis of necrotizing fasciitis in our patient was straightforward because it had evolved from an infected peripheral intravenous catheter site. Intravenous broad-spectrum antibiotics were administered; nevertheless, the patient’s phlebitis progressed to necrotizing fasciitis and to septic shock as clinically evident by his deteriorating mental status, hypotension, and decreased urine output.

As the patient’s sepsis progressed, he experienced respiratory distress, which may have been a result of leaky capillaries at the arterial-alveolar junction. Edema on alveolar cells changes their vital cell architecture because less surface area is available for effective gas exchange [5]. Impaired oxygenation, together with high oxygen requirements during a stressful septic period, may divert as much as 35–40% of blood flow to the diaphragm and respiratory muscles to keep up with the necessary oxygen demand [6]. Over time, ventilatory muscles fatigue and are unable to maintain a physiological acid–base status. Thus, a decision to intubate and provide mechanical ventilation was made to supply adequate oxygenation and ventilation while resting the patient’s ventilatory muscles. Sedation and pain control are also important aspects of care because they reduce anxiety, provide better ventilator-patient synchrony, reduce oxygen demand, and reduce the incidence of arrhythmias [7].

Throughout his septic period, the patient experienced an episode of hypotension that was unresponsive to fluid administration. With a massive “cytokine storm,” profound vasodilation and capillary leak occur, resulting in rapid fluid distribution into the interstitial space, thus leaving the intravascular space devoid of effective circulating volume and creating hypotension [4, 8]. Therefore, fluid resuscitation at this stage has the role of filling the intravascular volume, but its effects are transient because leaky capillaries eventually deplete the intravascular volume once again. In fact, after 90 minutes, less than 5% of infused fluid remains in the intravascular compartment of patients with sepsis [9]. Consequently, continuing with large-volume fluid administration with the hope of achieving adequate blood pressure and organ perfusion is associated with increased mortality.

In multiple studies of intravenous fluid resuscitation using either early goal-directed therapy or standard care, researchers have reported an average of more than 4 L of fluid administered during the initial 6 h of resuscitation [10–12], whereas fluid administered from 6 to 72 h averaged more than 8 L [13]. Our patient had received a total of 4 L of crystalloid infusion within 20 h in addition to 600 ml of bolus colloid infusion during his hypotensive episode. Nevertheless, his MAP remained inadequate, and 0.8 μg/kg/minute noradrenaline was required to meet an MAP above 65 mmHg. Additionally, his urine output had been less than 1 ml/kg/h for the previous 20 h along with a rising trend in serum creatinine.

At this point, the clinician needs to be wary in instituting further fluids because doing so will increase edema, especially to organs such as the liver and kidney. With progressive capillary leak, these encapsulated organs are unable to compensate for the increased volume, and severe interstitial edema will compress vital blood flow [14]. Aside from compression, increased edema leads to microvascular flow congestion and sluggish peritubular flow, as evidenced by our patient’s abrupt renal failure and positive fluid balance [15]. Hence, the use of early vasopressors is critical to maintaining an effective MAP necessary for adequate organ perfusion and to limiting edema formation [16].

We believe fluid therapy is best tailored to specific indications and that the administration of aggressive fluid administration should be restricted only to the resuscitation phase of septic shock. Hoste *et al*. best summed up the four stages of fluid therapy as divided into resuscitation, optimization, stabilization, and evacuation phases [17]. Our patient’s resuscitation phase began from day 1 of HCU care, when 4 L of normal saline were administered. As his hemodynamic status began to deteriorate, another 500 ml of colloid and 100 ml of 20% albumin were administered along with vasopressors to avoid hypoperfusion and correct uncompensated shock.

After the patient’s shock was managed, he entered the optimization phase, which is a state of compensated shock. At this phase, giving too little fluid will cause the patient to fall back into a shocked state, whereas giving too much fluid will cause inadvertent fluid overload [17]. Consequently, our patient was managed with 60 ml/h of fluid and vasopressor. After the optimization phase for 6 h in the ICU, repeat blood work revealed decreasing serum lactate and improving capillary refill time. However, it is noteworthy that increased lactate during sepsis is not a marker of tissue hypoxia, because lactate is produced as a result of adrenergic and inflammatory responses [18]. Hence, decreasing lactate in our patient marked the downregulation of the host inflammatory response, whereas improving capillary refill time and increasing urine output indicated improving organ perfusion.

Next comes the stabilization phase, which is the period when fluids are administered to provide daily requirements and replace ongoing losses, if any [3]. The Fluid and Catheter Treatment Trial (FACTT) researchers reported better outcomes for those patients who were treated using conservative fluid regimens than for those who received the usual “maintenance” fluids with liberal fluid management [19]. In this trial, patients who were treated with liberal fluid received an average of more than 4 L/day, as compared with those who underwent a conservative fluid regimen, who received 3.5 L/day, over the first week. Our patient received an average of 2.9 L/day, amounting from a normal saline infusion at 20–40 ml/h in addition to enteral nutrition and infusions of sedative, analgesic, antimicrobial, and vasoactive drugs.

In the evacuation phase of fluid therapy, fluids are deliberately removed from the patient, and the goal is to strive for a negative fluid balance [17]. In our patient, negative fluid balance was achieved through diuresis alone, without any drug intervention. Because his condition improved with appropriate antimicrobial therapy and a conservative fluid regimen, his renal function gradually improved by increasing urine output and subsequent lowering of urea and creatinine levels. However, at some point, the evacuation phase may involve the use of diuretics or renal replacement therapy with the goal of mobilizing excess fluid from the body.

Source control through wound debridement and necrotomy was performed, and 1 h postdebridement our patient experienced sudden hypotension that may have been due to toxic shock syndrome. Toxins liberated from *Streptococcus* during surgery spark an immunological response followed by massive release of histamine into the circulation, and they produce marked hypotension [4]. A bolus of 100 ml of normal saline was administered to rapidly fill the depleted intravascular volume, together with the institution of noradrenaline and epinephrine. The patient’s vital signs progressively stabilized, and within 2 h the epinephrine infusion was discontinued. Broad-spectrum antiobiotic therapy using meropenem, moxifloxacin, and fluconazole was administered while awaiting the results of culture and antibiotic sensitivity tests, which later revealed sensitivity to teicoplanin and moxifloxacin. Hence, both teicoplanin and moxifloxacin were administered starting on day 2 of ICU care, along with fluconazole because his sputum culture revealed growth of *Candida albicans*.

Another variable commonly sought in the management of patients with sepsis is the CVP. Throughout our patient’s care, his CVP value was not used as a guide in titrating fluid administration, because this method has been proven to be an unreliable method of measuring overall volume status. The heart and systemic vasculature is a complex system that functions together in preserving effective circulating volume by mobilizing fluids through vasoconstriction, vasodilation, or increasing cardiac output [20]. The response is dynamic and continually adapting at a rapid rate; thus, measuring static CVP at one point in the day to assess overall fluid status is inaccurate and ill-advised. In fact, values of CVP as well as intramural and pulmonary arterial occlusion pressure have been proven to have no correlation with circulating blood volume [21].

Aside from fluid input, an important aspect of improving clinical outcomes of patients with sepsis is to keep a watchful eye on their fluid balance. Cumulative fluid balance reported by critically ill nonsurvivors averaged 7761 ± 7391.9 ml for 1 week [2]. Our patient had a positive fluid balance during the first and second days of his ICU stay (+3540 ml and +1255 ml, respectively); nevertheless, he had a negative fluid balance on the following 4 days (−967 ml, −765 ml, −1998 ml, and −2537 ml, respectively) with a cumulative fluid balance of −1472 ml for 7 days in the ICU. His renal function improved; his mentation substantially improved; his respiratory distress quickly resolved; and his vasopressors were discontinued.

Throughout his ICU stay, our patient received normal saline and additional fluids from enteral nutrition at 1000 kcal/500 ml, which were given as intermittent 100-ml bolus feedings. Deep vein thrombosis prophylaxis was achieved through mechanical compression because thrombocytopenia, coagulopathy, and elevated D-dimer levels herald the development of disseminated intravascular coagulation and heparin was of limited use. Passive physiotherapy was initiated early to prevent muscle atrophy and stiffness, whereas active physiotherapy followed after the patient responded [22]. Bronchial hygiene and pulmonary toilet were exercised with daily mucolytic nebulizers, and suction was provided through a closed system device.

## Conclusions

Current evidence on fluids and sepsis urges us to reconsider the fluid regimen in the management of patients with sepsis because aggressive fluid administration after a state of resuscitated sepsis is well-documented to have the worst outcome. Patients with sepsis respond poorly to fluids because a massive and erratic cytokine storm results in arteriovenodilation and microcirculatory dysfunction during the early stages of septic shock; hence, fluid administered during the resuscitation phase is best given with vasopressors and early. After this phase, fluids must be tapered to prevent inadvertent fluid overload, which will worsen oxygen transport at the cellular level. Successful management of sepsis requires an integrated approach of infection control, use of appropriate antimicrobials, and supportive care. But perhaps every clinician ought to be extra vigilant in prescribing the most routine drug of all, fluids.
